# Comparative Filtration Performance of Composite Air Filter Materials Synthesized Using Different Impregnated Porous Media

**DOI:** 10.3390/ma16134851

**Published:** 2023-07-06

**Authors:** Yuxia Zeng, Qing Liu, Xin Zhang, Zhao Wang, Tao Yu, Fei Ren, Puchun He

**Affiliations:** 1 School of Resources Engineering, Xi’an University of Architecture and Technology, Xi’an 710055, China; 15309000205@163.com (Y.Z.); zhangxin17@xauat.edu.cn (X.Z.); wangzhao@xauat.edu.cn (Z.W.); 2 Wuhan Second Ship Design and Research Institute, Wuhan 430205, China; 3 XAUAT Engineering Technology Co., Ltd., Xi’an 710055, China; jkdgcjs@xauat.edu.cn; 4 Yan’an Branch of Shaanxi Provincial Land Engineering Construction Group Co., Ltd., Yan’an 716000, China; hepuchun123@163.com

**Keywords:** porous materials, compound materials, fiber structures, filtration performance, comparative analysis

## Abstract

Indoor environment quality is currently a hot research topic. In this study, composite air filter materials were synthesized using different impregnated porous medium materials, and their filtration performance and structural parameters were analyzed. The results showed that composite filter materials’ structures changed at the fibers’ surfaces when synthesized using different porous medium material layers. The filtration efficiency of composite filter materials synthesized using different porous media reached a maximum 0.8 m/s filtration velocity, and PM_10_, PM_2.5_, and PM_1.0_ increased by 1.67~26.07, 1.19~26.96, and 1.10~21.98%, respectively. The filtration efficiencies of reduced graphene oxide composite for PM_10_, PM_2.5_, and PM_1.0_ were 21.26, 20.22, and 18.50% higher, respectively, than those of carbon black composite. In addition, the filtration efficiency of the composite material synthesized by reducing graphene oxide improved for 0 to 1.0 μm particulates and was more effective by comparison. Filtration efficiency and resistance were comprehensively considered during air filter use to provide useful values for the selection and preparation of composite filter materials in the future.

## 1. Introduction

High concentrations of particulate matter are inconvenient during transportation and for people’s physical and mental health [1,2], particularly respiratory diseases, etc. [3,4]. Air pollutants are mostly complex pollutants consisting of all particle types, including harmful gases, bacteria, viruses, etc. [5,6]. The relevant literature indicates that harmful gases and bacteria might bind with particulate matter, which is significantly more harmful [7]. In addition, people pay more attention to indoor environments in the post epidemic era [8]. Therefore, how to create a healthy and safe environment will be a significant priority for current research.

Air filters have been widely used in the construction of indoor environments [9]. Scholars from different countries have researched filtration efficiency [10], filter structures [11], filter materials’ costs [12], and new materials [13,14,15] in many related studies [10,11,12,13,14,15]. However, due to the changing environment and increasing demand, research regarding new materials is a primary direction of current research [13,14,15]. The new materials’ research cycle is relatively long, with a complex preparation process and high economic cost [15], and the new materials’ stable performance cannot be guaranteed. Therefore, it is difficult to widely promote new filter materials at this stage in their development.

At present, optimizing and designing existing fiber filter materials are relatively mature and common technical processes [16,17]. The common method uses porous medium characteristics and an organic combination of existing materials to prepare new composite materials [18,19,20] and to manufacture a multi-function composite. Related synthesis technologies include electrospinning [21], impregnation synthesis [15], spraying [22], etc. However, at present, impregnation synthesis technology is widely used due to factors such as the preparation process and cost. Commonly used raw porous materials include carbon black [17,23], graphene [24], graphene oxide [25], and carbon nanotube [26].

It is well known that carbon black is easily obtained from nature and extremely stable [17]. It does not easily react with other substances and is widely used as a raw material for synthetic composite materials. At present, the relevant literature includes studies of carbon black synthetic filter materials [15,17], carbon black masks, etc. Based on these carbon black studies, graphene has been applied in the environmental protection field, specifically to large surface areas [27,28,29]. In-depth research has also been carried out regarding graphene synthetic filter materials [27], masks [28], composite materials [29], etc.; it has relatively broad applications. However, there are few comparative studies of composite materials synthesized using carbon black materials and reduced graphene oxide materials; research regarding how filter materials’ structural parameters and filtration performances are improved using different porous materials is even more insufficient. In addition, synthesized porous materials’ filtration performance is unstable, and experimental results often differ from actual application results. Especially in the post-epidemic era, air filters need to be replaced more frequently in large indoor areas. As a result, air filters’ overall performance requirements will steadily increase compared with past requirements.

Therefore, this study addressed the practical problems noted above. The filtration performance and structural parameters of composite air filter materials impregnated with different porous medium materials were tested and analyzed to provide useful support for synthesis research and the development of filter materials.

## 2. Methods

### 2.1. Materials

According to previous market research, polyester and non-woven fabric are currently the two materials used in combined air filters [15,17]; non-woven fabric is more widely used as a two-stage combination material for end filtration. Therefore, the most commonly used non-woven fabric material was selected as this study’s background material [15,17]. The impregnation method is suitable for materials with small porosity but not for polyester materials [15]. The direct impregnation method was used to prepare this study’s composite air filter materials using different porous media [15,17].

**Figure 1:**
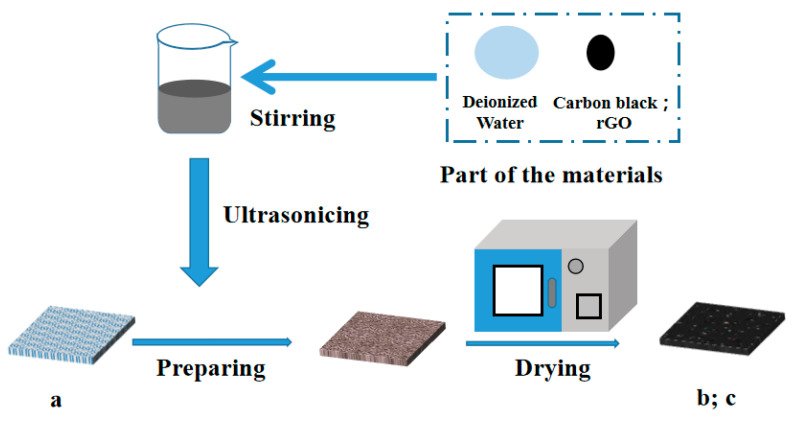
Schematic of an experimental operation. a, blank group; b, carbon black synthetic material; c, reduced graphene oxide material.

Three pieces of 5 cm × 5 cm non-woven fabric (F6, certification EN779, ISO9001) from the same batch and model were selected as background materials (GuangDong Fresh Filter Co. Ltd., Foshan, China) [15,17]. The first piece (marked a) was the blank group. The second piece was carbon black synthetic material (Changzhou Yongguang Material Co. Ltd., Changzhou, China) [17], Shen Ling brand, model C311 [17]; its specific surface area was 200–400 m^2^/g. The carbon black was dissolved, stirred, and ultrasonically treated. Next, the background material was impregnated and dried to synthesize carbon black synthetic material (marked b). Refer to the specific experimental steps described in [15,17]. The third piece was reduced graphene oxide material. The graphene oxide, an industrial-grade material (Suzhou Tanfeng Graphene Technology Co. Ltd., Suzhou, China), was dissolved, stirred, and ultrasonically treated. Next, the background material was soaked and dried for synthesis. The synthesized material was placed in the reducing agent in a water bath, heated, and then dried to obtain the reduced graphene oxide material (marked c). The novelty of this study is primarily the process used to synthesize and prepare new materials. As is well known, small changes in relevant factors during the preparation process can cause significant differences in the parameters of synthesized materials; these differences can cause significant changes in the subsequent comprehensive performance indicators. The specific parameter settings were obtained through experimental optimization; Figure 1 shows the preparation flow chart.

### 2.2. Experimental Systems

**Figure 2:**
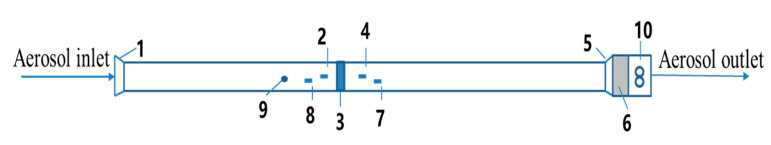
Experimental system. 1, current collector; 2, upstream static pressure probe; 3, tested filter; 4, downstream static pressure probe; 5, expanding tube; 6, soft connection; 7, downstream sampling head; 8, upstream sampling head; 9, wind speed hole; 10, axial fan.

A GRIMM1.109 portable aerosol spectrometer was used to measure concentrated particles before and after the filters were used [15,17]. The concentration count limit was 2,000,000 P/L [15,17]. There were 31 channels (from 0.25 to 32 μm particles) with 5% repeatability. A HD2114P.0 portable micromanometer was used to measure filter resistance [15,17], with a ±2% reading + 0.1 m/s measuring accuracy [15,17] and ±0.4% F.S. pressure range. A HD37AB1347 indoor air quality monitor was used to measure velocity, with a ±3% accuracy range. To reduce errors, 5-min average concentrations were recorded before and after the test filter [15,17]. Figure 2 shows the experimental system used to assess filtration performance, which, along with the measuring points indicated, was designed according to China’s standards [30,31].

### 2.3. Air Filter Performance

Filtration efficiency can be calculated using Equation (1) [17]:

(1) $$\eta =\frac{C_{1}-C_{2}}{C_{1}}\times 100\%$$

where *η* is filtration efficiency (%) [17]; *C*_1_ is the concentration of particulates before filtration (μg/m^3^) [17]; and *C*_2_ is the concentration of particulates after filtration (μg/m^3^) [17].

Filtration resistance can be calculated using Equation (2) [17]:

(2) $$\Delta P=P_{2}-P_{1}$$

where *P*_1_ is resistance before filtration (Pa) [17], and *P*_2_ is resistance after filtration (Pa) [17].

## 3. Results and Discussion

### 3.1. SEM of Different Air Filters

**Figure 3:**
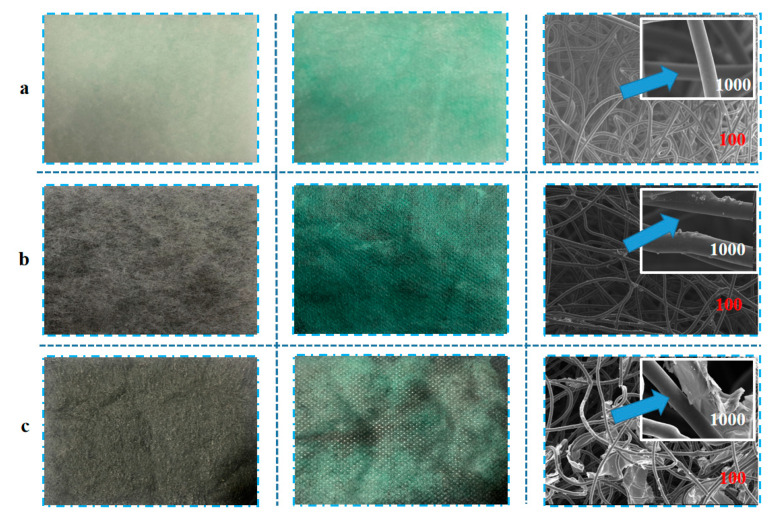
SEM of different air filter materials.

SEM of blank control group materials (a), carbon black synthetic materials (b), and reduced graphene oxide materials (c) with 100- and 1000-times magnification are shown in Figure 3.

Figure 3 shows that under 100-times SEM, fabric a’s surface was smooth and naturally twisted. Under 1000-times SEM, fabric a’s surface was relatively orderly and not rough [17]; the structure between fibers was loose with large porosity. After synthesis, the surface fibers of fabrics b and c appeared rough and showed obvious processing under 100-times SEM; the entire fabric surface contained a coating layer composed of different porous media. Additionally, a thin sheet connection phenomenon appeared between the fabric c’s fibers.

Under 1000-times SEM, a massive accumulation phenomenon was visible in fabric b’s carbon black coating, and the carbon black was cross-linked. The coating had many folds, and the surface was rough, which was reported in previous studies [17]. Under 1000-times SEM, blocky accumulation accompanied by the effect of fiber conjunctiva was visible in fabric c’s coating layer of reduced graphene oxide. The main reason for this is that graphene is a porous medium. During the preparation process, reduced graphene oxide filled gaps between the fibers, which were prone to beading and the spindle phenomenon [32]; this verified this study’s correctness by showing that the composite air filter materials impregnated with different porous media changed the fibers’ diameters, filling ratios, porosities, fiber mechanical strengths, and other material parameters, as shown in Table 1.

### 3.2. Particle Concentrations in the Atmosphere

Atmospheric particles were tested [33]; particle distributions are shown in Table 2.

According to Table 2’s data, particles from 0 to 2.5 μm accounted for the majority of the atmosphere (approximately 99.88%); particles from 0 to 1.0 μm accounted for 99.55%. The atmospheric particles in Xi’an were mainly small particles; this was consistent with conclusions in relevant research [34]. These particles increase health risks [35]; thus, improving air filters’ purification effect is urgent.

### 3.3. The Influence of Filtration Velocity

**Figure 4:**
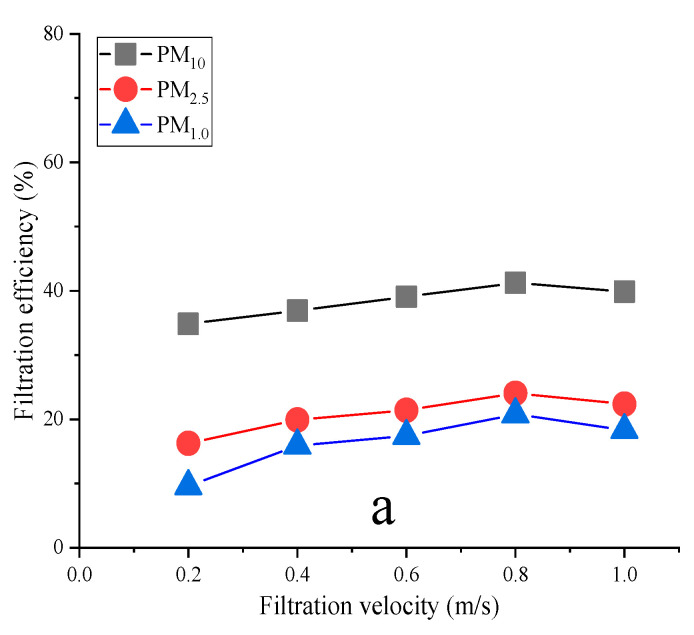

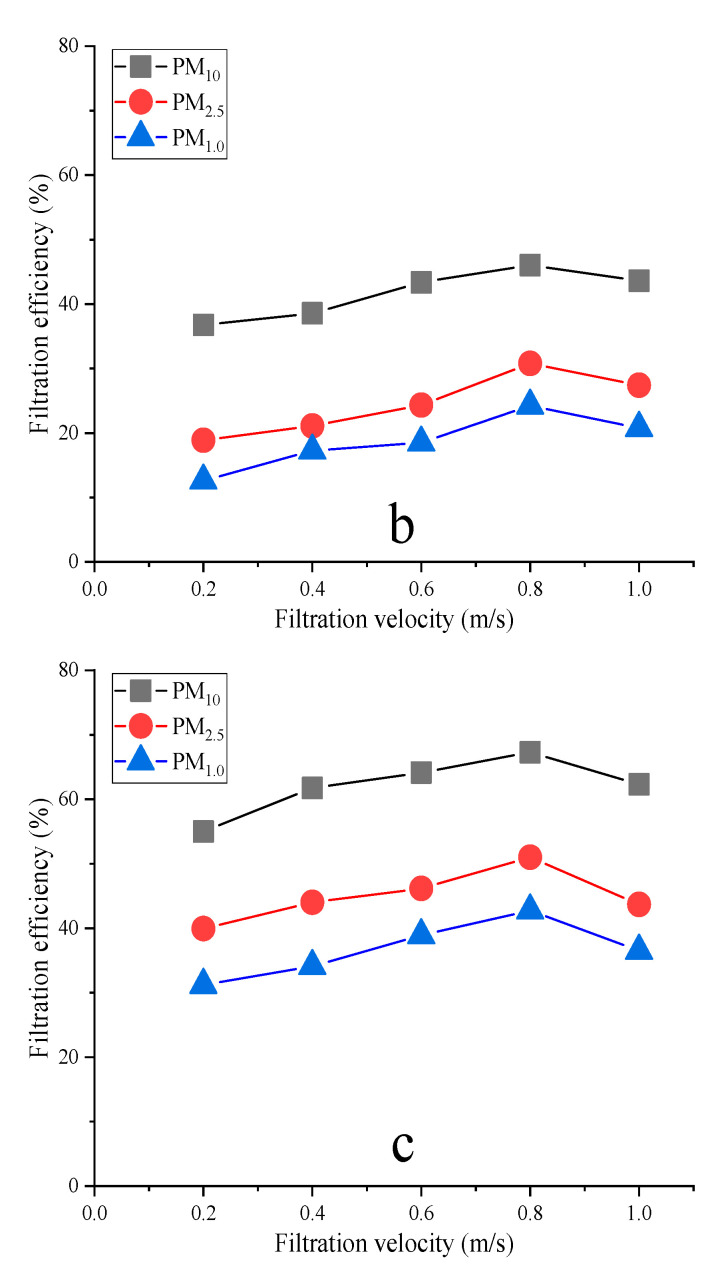
The trend in filtration efficiency changed with filtration velocity. (**a**), blank group; (**b**), carbon black synthetic material; (**c**), reduced graphene oxide material.

The variation in filtration efficiency with filtration velocity for three materials is shown in Figure 4.

Figure 4 shows that the three materials’ filtration efficiency first increased and then decreased as filtration velocity increased. Fabric a’s filtration efficiency for PM_10_ was 34.84~41.23%, for PM_2.5_ it was 16.26~24.06%, and for PM_1.0_ it was 9.58~20.77%. Fabric b’s filtration efficiency for PM_10_ was 36.78~46.03%, for PM_2.5_ it was 18.87~30.79%, and for PM_1.0_ it was 12.62~24.25%. Fabric c’s filtration efficiency for PM_10_ was 54.99~67.29%, for PM_2.5_ it was 39.94~51.01%, and for PM_1.0_ it was 31.21~42.74%.

**Figure 5:**
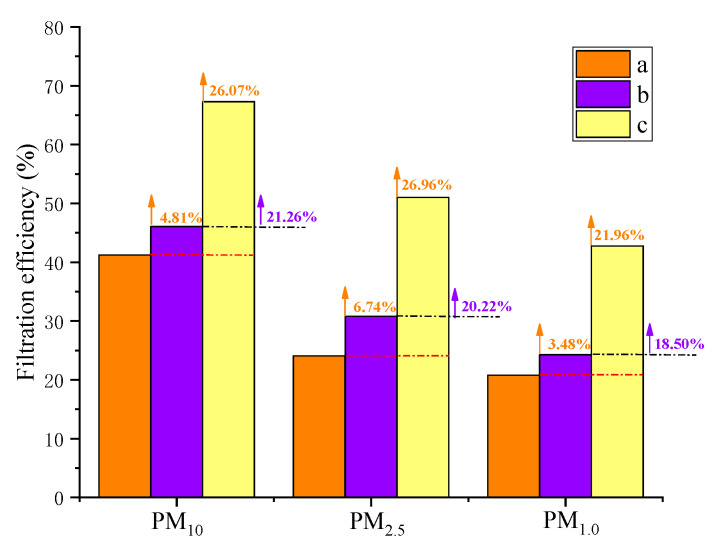
PM differences in three porous medium composite filter materials.

The filtration efficiencies of fabrics a, b, and c for PM_10_ were 41.23, 46.03, and 67.29%, respectively; for PM_2.5_ they were 24.06, 30.79, and 51.01%, respectively; and for PM_1.0_ they were 20.77, 24.25, and 42.74%, respectively. The filtration efficiencies of the synthesized porous composite materials (b and c) for PM_10_, PM_2_._5,_ and PM_1.0_ increased by 1.67~26.07%, 1.19~26.96, and 1.10~21.98%, respectively, compared with those of the blank control group material (a). The reduced graphene oxide composite filter material (c) had a better and more obvious purification effect, as seen in Figure 4. Differences in filtration performance at the optimal filtration velocity of composite filter materials synthesized using different porous media are shown in Figure 5.

Figure 5 shows that the filtration performances of the composite material synthesized using reduced graphene oxide were better than those of the composite material synthesized using carbon black. Fabric c’s filtration efficiency for PM_10_ was 21.26% higher than that of fabric b. Fabric c’s filtration efficiency for PM_2.5_ was 20.22% higher than that of fabric b. Fabric c’s filtration efficiency for PM_1.0_ was 18.50% higher than that of fabric b. Brownian motion affects small particles [36]. The improved fabric c had relatively small porosity. The collision rate between particles and fibers increased under the effects of inertia and interception. As a result, fabric c’s efficiency for PM_1.0_, PM_2.5_, and PM_10_ obviously improved.

In addition, reduced graphene oxide molecules provided active sites for particle adsorption [32]. This increased the number and strength of active sites while reducing the material’s porosity and increasing the pollutants’ contact area [37]. Its porous structure ensured airflow while the surface forced the pollutants to adsorb, thus improving particle capture capacity, reducing particle escape, and further increasing the particle capture effect.

### 3.4. Counting Efficiency of Different Particles

**Figure 6:**
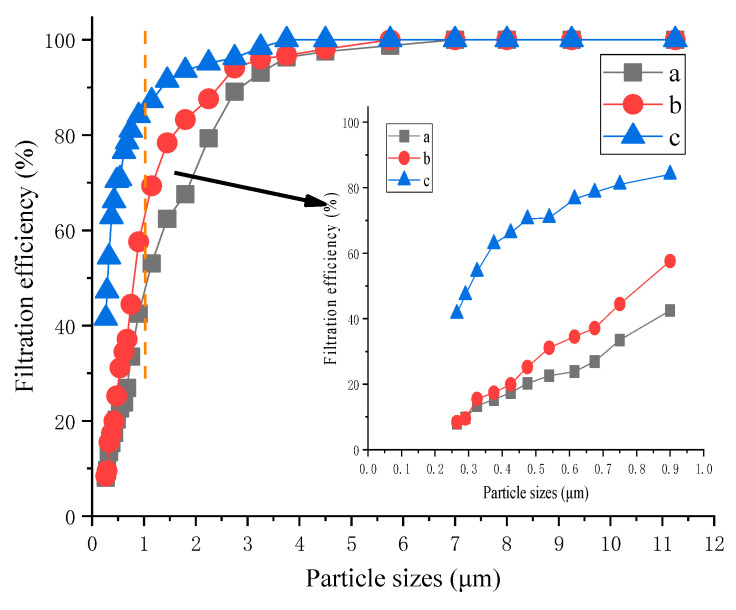
Counting the efficiencies of fabrics a, b, and c at optimal velocity by particle size.

The counting efficiencies of composite filter materials synthesized using different porous media at the optimal filtration velocity are shown in Figure 6.

Figure 6 shows that as particle size increased, the counting efficiency of the porous medium composite filter materials increased; the filtration performance of the composite material synthesized using reduced graphene oxide was better than that of the composite material synthesized using carbon black. Fabric b’s counting efficiency was 0.33~16.32% higher than that of fabric a. Fabric c’s counting efficiency was 1.20~50.17% higher than that of fabric a. Fabric c’s counting efficiency was 1.94~46.24% higher than that of fabric b.

The filtration performances of different porous medium composite filter materials significantly varied for 0.6 to 2.5 μm particulates; fabric b’s filtration was 18.86~46.24% below fabric c’s for 1.0 μm particles because Brownian motion significantly affected 1.0 μm particles [38], and small particles move easily in fibers. The porosity of the composite fiber decreased after the synthesis of reduced graphene oxide, which increased the particle capture efficiency under the effects of inertia and interception [39]. Therefore, the composite synthesized using reduced graphene oxide had a better purification effect for particles between 0 and 1.0 μm. However, filtration efficiencies for large particles followed similar trends in all fabrics [40].

### 3.5. The Influence of Filtration Resistance

**Figure 7:**
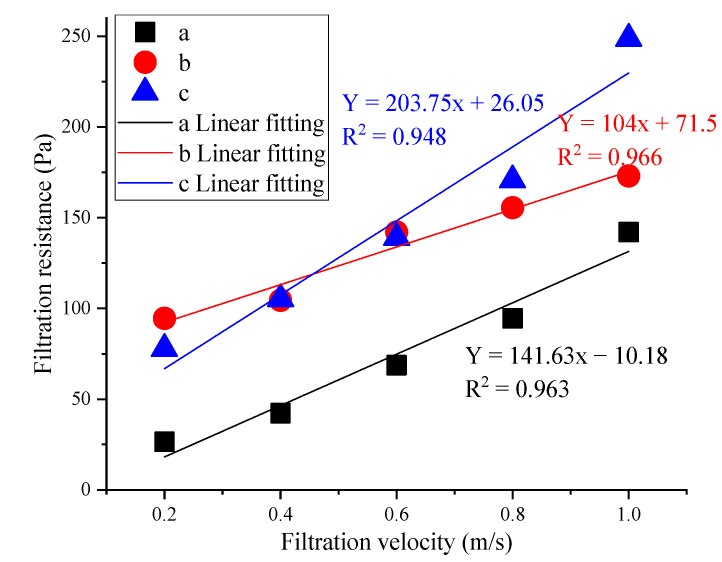
Initial resistance of filter materials using varied filtration velocities.

The relationship between initial resistance and filtration velocity is shown in Figure 7.

Figure 7 shows the trend of increasing filtration velocity and resistance of different porous medium composite filter materials with a good fitting effect [20]. Fabric a’s resistance range was 26.5 to 142.0 Pa, fabric b’s was 94.5 to 173.0 Pa, and fabric c’s was 78.0 to 249.0 Pa. Fabric b’s resistance was 31.0~73.0 Pa higher than that of fabric a; fabric c’s resistance was 51.0~107.0 Pa higher than that of fabric a and 1.0~76 Pa higher than that of fabric b.

The synthesized material’s resistance range was much higher than that of the raw material; the porosity of the synthesized filter material became smaller, which affected the airflow’s uniformity [15]. In addition, the increase in the synthesized fiber’s roughness led to an increase in friction resistance between particles and the fiber surface, which led to a corresponding increase in the fiber’s resistance [40]. The resistance of fabric c was much greater than that of fabric b, mainly because gas flowing through material with a small porosity encountered greater resistance than gas flowing through material with a large porosity. Therefore, it is necessary to match resistance with filtration efficiency during actual use [41]. With China’s continuous development of deep mining areas, composite materials will have more widespread applications in underground mining environments [42,43].

## 4. Conclusions

The filtration performances and structures of composite air filter materials impregnated with different porous medium materials were studied and analyzed in this study. The conclusions are as follows:

1. Synthesizing composite filter materials using different porous media changed the materials’ fiber diameters, filling rates, porosities, and other parameters and introduced a coating layer phenomenon. The carbon black coating layer had a massive accumulation phenomenon. The reduced graphene oxide coating layer also had a massive accumulation phenomenon as well as fiber bonding.
2. The filtration of composite filter materials synthesized using different porous media reached maximum efficiency at an 0.8 m/s filtration velocity. The filtration efficiencies of composite filter materials for PM_10_, PM_2.5_, and PM_1.0_ increased by 1.67~26.07%, 1.19~26.96%, and 1.10~21.98%, respectively. Fabric c’s filtration efficiency for PM_10_ was 21.26% higher than that of fabric b; fabric c’s filtration efficiency for PM_2.5_ increased by 20.22% compared with fabric b’s; and fabric c’s filtration efficiency for PM_1.0_ was 18.50% higher than that of fabric b. The composite filter material made of reduced graphene oxide after synthesis had better overall filtration efficiency.
3. For 0 to 1.0 μm particles, the difference in filtration efficiency between fabrics c and b was 18.86~46.24%. The composite material synthesized using reduced graphene oxide had improved filtration for 0 to 1.0 μm particulates, mainly due to its fiber structure and porosity.
4. Filtration efficiency and resistance should be taken into account during use. This study provides useful values for the preparation and optimization of composite filtration materials in later stages of development.

Through experimentation, the porous composite prepared by reducing graphene oxide was found to be more effective. The next step is to conduct in-depth research regarding the graphene oxide composite material’s specific preparation process and other performance parameters, such as adsorption of harmful gases, disinfection, sterilization of microorganisms, etc. This study provides basic data for the protection of public buildings’ indoor environments.

## Figures and Tables

**Table 1:** Relevant parameters of air filter materials.

Sample	Size (cm^2^)	Fiber Diameter (μm)	Filling Rate (%)	Porosity (%)
a	25.00	20.01 ± 0.02	2.19 ± 0.02	97.81 ± 0.02
b	25.00	34.82 ± 0.05	4.65 ± 0.03	95.35 ± 0.03
c	25.00	22.00 ± 0.03	5.50 ± 0.02	94.50 ± 0.02

**Table 2:** Concentration distributions of atmospheric particulates.

The Range of Particles (μm)	The Average of Particles (μm)	Quantity Proportion (%)
0~0.5	0.25	94.75
0.5~1.0	0.75	4.80
1.0~2.5	1.75	0.34
2.5~5.0	3.75	0.09
5.0~10	7.5	0.01
10~30	20	0.001

Note: The average temperature was 17.2 °C~26.8 °C, and the average humidity was 36.1%~52.3%.
